# Dr. Russ; Associate Editor of the “Red-Back”

**Published:** 1901-04

**Authors:** 


### TEXAS MEDICAL JOURNAL.

AUSTIN, TEXAS.

A MONTHLY JOURNAL OF MEDICINE AND SURGERY.

EDITED AND PUBLISHED BY
F. E. DANIEL, M. D.

ASSOCIATE EDITOR:
WITTEN BOOTH RUSS,
San Antonio, Texas.

Published Monthly at Austin, Texas. Subscription price $1.00 a year in advance.

Eastern Representative: John Guy Monihan, St. Paul Building, 220 Broadway, New York City.

Official organ of the State Association of Health Officers, the West Texas Medical Association, the Houston District Medical Association, the Austin District Medical Society, the Brazos Valley Medical Association, the Galveston County Medical Society, and several others.

### DR. RUSS; ASSOCIATE EDITOR OF THE "RED-BACK".

The JOURNAL takes pleasure in introducing to its readers and the medical profession at large Dr. Witten Booth Russ, of San Antonio, who will supplement the editorial work of the "Red-Back" in future, having especial charge of the book reviews and the exchanges. Publishers are requested to send to Dr. Russ, direct, books intended for review in the JOURNAL, and our "esteemed contemporaries" are requested, without further notice, to send our exchange copies to him, No. 3 Hicks Building, San Antonio, Texas. Dr. Russ is an *alumnus* of the Medical Department of the University of Pennsylvania. He was Assistant Surgeon to the National Soldiers' Home, at Hampton, Va., from July, 1898, to January, 1899, and from that time till October, 1900, was physician in charge of Girard College, a position won by competitive examination in a contest with over thirty competitors. As such he had medical and sanitary charge of 1600 boy students and 400 employes. Dr. Russ was married in Philadelphia, in July, 1900, to Miss Jean McGrath of that city, a ward of Col. A. K. McClure, the well-known publisher, and editor of the *Philadelphia Times*. Dr. Russ is a thoroughly up to date physician and has already done good JOURNAL work.

The Association of Texas Health Officers will meet at Dallas, August 2nd. Every health officer in the State should be present. There are 246 counties in the State and each county should be represented. Besides these 246 physicians who should all promptly join the association, every city, town and village has its health officer, and there is no reason why one thousand should not be enrolled at the Dallas meeting. There are immense possibilities in such an organization. By unanimity of action on the part of these sanitary officers doubtless the State can be induced at next session of the Legislature to create a State Board of Health—an organization which shall take cognizance of other matters than smallpox and yellow fever, in place of the very primitive, obsolete and utterly inadequate "quarantine department," the inability of which to even deal with smallpox has been recently strikingly demonstrated.

